# Untargeted Lipidomics Analysis of the Cyanobacterium *Synechocystis* sp. PCC 6803: Lipid Composition Variation in Response to Alternative Cultivation Setups and to Gene Deletion

**DOI:** 10.3390/ijms21238883

**Published:** 2020-11-24

**Authors:** Weronika Hewelt-Belka, Ágata Kot-Wasik, Paula Tamagnini, Paulo Oliveira

**Affiliations:** 1 Department of Analytical Chemistry, Faculty of Chemistry, Gdańsk University of Technology, Gabriela Narutowicza 11/12, 80-233 Gdańsk, Poland; werbelka@pg.edu.pl (W.H.-B.); agawasik@pg.edu.pl (Á.K.-W.); 2 i3S—Instituto de Investigação e Inovação em Saúde, Universidade do Porto, R. Alfredo Allen 208, 4200-135 Porto, Portugal; pmtamagn@ibmc.up.pt; 3 IBMC—Instituto de Biologia Molecular e Celular, Universidade do Porto, R. Alfredo Allen 208, 4200-135 Porto, Portugal; 4 Departamento de Biologia, Faculdade de Ciências, Universidade do Porto, R. Campo Alegre s/n, 4169-007 Porto, Portugal

**Keywords:** cyanobacteria, untargeted lipidomics, cultivation setup, genetic engineering, lipid metabolism

## Abstract

Cyanobacteria play an important role in several ecological environments, and they are widely accepted to be the ancestors of chloroplasts in modern plants and green algae. Cyanobacteria have become attractive models for metabolic engineering, with the goal of exploring them as microbial cell factories. However, the study of cyanobacterial lipids’ composition and variation, and the assessment of the lipids’ functional and structural roles have been largely overlooked. Here, we aimed at expanding the cyanobacterial lipidomic analytical pipeline by using an untargeted lipidomics approach. Thus, the lipid composition variation of the model cyanobacterium *Synechocystis* sp. PCC 6803 was investigated in response to both alternative cultivation setups and gene deletion. This approach allowed for detecting differences in total lipid content, alterations in fatty-acid unsaturation level, and adjustments of specific lipid species among the identified lipid classes. The employed method also revealed that the cultivation setup tested in this work induced a deeper alteration of the cyanobacterial cell lipidome than the deletion of a gene that results in a dramatic increase in the release of lipid-rich outer membrane vesicles. This study further highlights how growth conditions must be carefully selected when cyanobacteria are to be engineered and/or scaled-up for lipid or fatty acids production.

## 1. Introduction

Cyanobacteria are a remarkably diverse group of prokaryotes, with wide ecological distribution, great metabolic plasticity, and a high impact on Earth’s carbon and nitrogen geochemical cycles. Their capacity of using sunlight to power metabolic processes and binding chemical energy has attracted enormous attention. In the last few years, synthetic biology and metabolic engineering initiatives have been engaged in developing these photosynthetic prokaryotes as robust and sustainable microbial cell factories, particularly *Synechocystis* sp. PCC6803, considered by some as the “Green *E. coli*” [1]. In this context, fatty acid biosynthesis (e.g., [2,3]) and lipid production (e.g., [4,5,6]) in *Synechocystis* sp. PCC 6803 have been the focus of alternative approaches from various laboratories with the common goal of improving production titers, envisioning cyanobacteria as potential sources for third generation biofuels. In addition, lipid analysis has also been a topic of interest over the years to elucidate adaptations of *Synechocystis* sp. PCC 6803 to environmental factors, particularly changes in temperature [7,8], but also because lipids are key molecules in primary metabolism, especially in the light reactions of photosynthesis [9,10,11]. More recently, lipidomics approaches have also been used to analyze and compare lipid composition of *Synecchocystis* sp. PCC 6803 in response to changes in other environmental conditions [12,13] and upon deletion of genes that encode key proteins in several cellular and metabolic processes [14,15].

As compared to lipids in algae, plant, and animal tissues that are mainly triacylglycerols, cyanobacterial lipids are rather different, as they are mostly composed of diacylglycerols (DG) [16]. In addition, due to ultrastructural and physiological traits, DG-based lipids in cyanobacteria are mainly located in thylakoid membranes, the photosynthetic membranes, the largest membrane system in the cell. In agreement, lipid components of cyanobacteria are conserved with those of the chloroplast membranes, mainly composed of neutral galactolipids (monogalactosyldiacylglycerol (MGDG) and digalactosyldiacylglycerol (DGDG)) and two types of anionic lipids (sulfoquinovosyldiacylglycerol (SQDG) and phosphatidylglycerol (PG)) [17,18]. Because photosynthesis is so prevalent, the major glycerolipid, MGDG, is the most abundant membrane lipid on Earth [19]. Moreover, within a specific lipid class, cyanobacterial lipid species can vary in the number of carbon atoms in fatty acyl substituent and level of saturation [17].

Several methods have been investigated to optimize lipid extraction from the cyanobacterium *Synechocystis* sp. PCC 6803 (e.g., [16]), which have also been coupled to various techniques to study its lipid composition. These include easy ambient sonic-spray ionization mass spectrometry (EASI-MS) [13], electrospray ionization tandem mass spectrometry (ESI-MS/MS) [20], and even estimating lipid quality and quantity by converting lipid species previously separated by thin-layer chromatography to fatty acid methyl esters (FAMEs) via acid-catalyzed trans-esterification [16]. Nevertheless, while the analytical tools to study DNA, RNA, and proteins are quite robust, lipid analysis in cyanobacteria still lags far behind. Technical advances in high-performance liquid chromatography-mass spectrometry (HPLC-MS) have allowed to shorten the analysis time and detection sensitivity of a variety of lipid species. In fact, one of the main approaches in mass spectrometry (MS)-based workflows is referred to as untargeted or discovery lipidomics. This strategy involves unbiased qualitative and quantitative analysis of a lipidome by analyzing an entire lipid extract by either liquid chromatography (LC)-MS or direct infusion-MS without prerequisite targets. Untargeted platforms are unbiased and have broad coverage, making it possible to analyze several hundreds to thousands of individual lipid species present in a sample. This approach is now widely applied to detect and to characterize lipidomic changes between bacterial strains [21,22,23].

In this work, we decided to expand the lipidomic analytical pipeline of cyanobacteria. The model cyanobacterium *Synechocystis* sp. PCC 6803 was grown in photoautotrophic conditions, under two different cultivation setups, fast- and slow-growth conditions, which result in different doubling times and induce specific physiological adaptations. In addition, the mutant strain lacking a functional TolC protein [24], an outer membrane protein involved in the secretion of a number of different molecules, ranging from proteins to antibiotics [24,25,26] and also fatty acids [27], was also included for analysis. Untargeted lipidomics analysis revealed significant differences in lipid content when the strains were cultivated in different conditions, while the genetic deletion of *tolC* resulted in minor differences. These results help broadening the cyanobacterial lipidomics analytical platforms and strongly support the notion that different cultivation setups and conditions have major impacts in cyanobacterial cells lipid composition, and thus in lipid metabolism. The realization of these differences will help in optimizing cyanobacterial cultivation conditions to match with lipid production strategies.

## 2. Results and Discussion

### 2.1. Different Photoautotrophic Cultivation Setups Result in Marked Differences in Cyanobacterial Growth Rates

**Figure 1:**
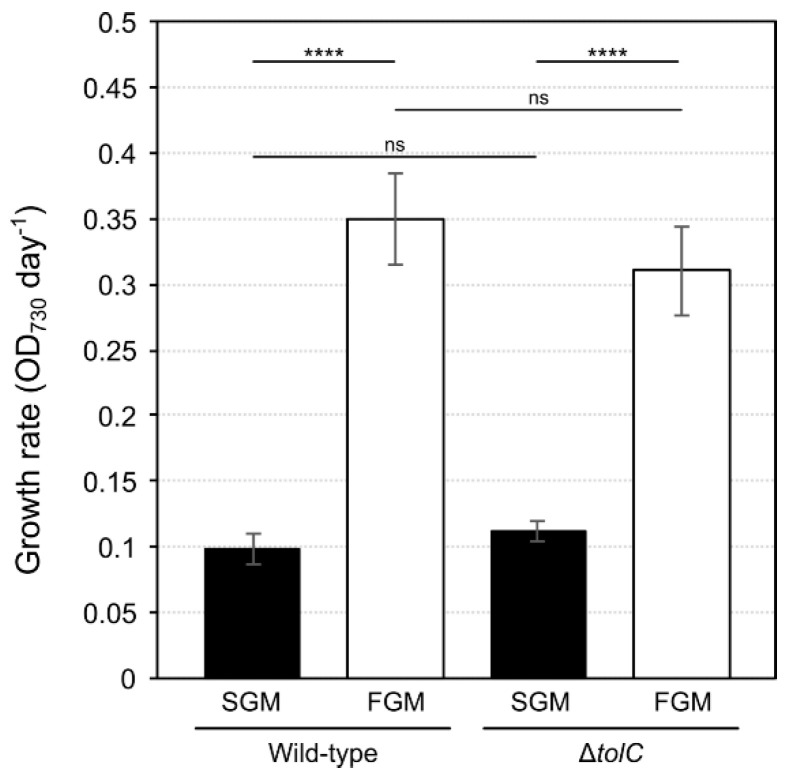
Growth rates of *Synechocystis* sp. PCC 6803 wild-type and Δ*tolC* mutant strains cultivated in slow- (SGM, black bars) or fast-growth mode (FGM, white bars). The presented rates were determined based on the respective culture’s initial and final optical densities (OD_730_), and on for 4 days of cultivation. The error bars indicate standard deviation corresponding to 6 independent biological replicates. **** *p* < 0.0001; ns: not statistically significant difference.

*Synechocystis* sp. PCC 6803 is a photoautotrophic organism, whose growth is dependent on the presence of light. Even when the growth medium is supplemented with glucose and cells are forced to grow heterotrophically in darkness, a daily light-pulse of approximately 5 min is absolutely necessary to sustain growth (the so-called light-activated heterotrophic growth (LAHG) [28]). Here, we tested two widely used (e.g., [24,29]), and yet alternative photoautotrophic cultivation setups that result in major differences in growth. In both conditions, light intensity and periodicity, temperature, and medium composition were the same. However, in slow-growth mode (SGM), the culture was inoculated to an initial optical density at 730 nm (OD_730_) of 0.1 and maintained in Erlenmeyer flasks rotating in an orbital shaker, while in fast-growth mode (FGM) cultures were inoculated to an initial OD_730_ of 0.05 and bubbled with air in glass gas-washing bottles, both of which cultivated for 4 days. *Synechocystis* sp. PCC 6803 wild-type cultures showed growth rates of approximately 0.1 OD_730_ day^−1^ in SGM, and of about three-fold higher in FGM (Figure 1). Under the tested conditions, the photoautotrophic cyanobacterium *Synechocystis* sp. PCC 6803 showed a linear growth, and not the typical exponential growth of heterotrophic bacteria. Four days of cultivation allowed cells in both cultivation setups to undergo at least two division cycles, making sure that cells were physiologically adapted to the cultivation setup. Moreover, the time of cultivation also proofed sufficient to overcome eventual differences resulting from different initial densities, while resulting in enough biomass that could be further processed for downstream analyses. More efficient gas transfer/gas exchange and mixing of the culture in FGM are two possible reasons that can account for the differences observed in growth rates [30,31].

### 2.2. Synechocystis sp. PCC 6803 Lipidomic Variations in Cells Cultivated in SGM and in FGM

Lipid composition has long been shown to vary in a number of organisms depending on growth conditions (e.g., [32,33,34,35]). In *Synechocystis* sp. PCC 6803 lipid composition was also found to be significantly different when comparing cells cultivated in mixotrophic (photoautotrophy and supplemented with glucose) and in LAHG conditions [20] or under nitrogen starvation [13]. We used an untargeted lipidomic analytical pipeline to evaluate comprehensively the differences in lipid composition of *Synechocystis* sp. PCC 6803 samples obtained after cultivation in SGM and in FGM, i.e., in photoautotrophy, but under conditions that result in significant differences in growth rate between each other.

*Synechocystis* sp. PCC 6803 is a Gram-negative bacterium with high degree of cross-linking between peptidoglycan chains [36], resulting in resistance to organic-solvent penetration, which can hinder the release of cellular lipids during the extraction steps [16]. An appropriate sample preparation is therefore crucial for the effective extraction of lipids resulting in high lipidome coverage. We applied liquid-liquid extraction technique based on chloroform:methanol:water solvent system with the addition of glass beads to facilitate cell disruption and lipid release. Subsequently, diluted lipid extracts were analyzed in an untargeted approach with the use of reversed-phase liquid chromatography–quadrupole-time of flight mass spectrometry (RPLC-Q-TOF-MS) in positive ionization mode. The analytical workflow used in this study is presented in Figure S1. Chromatographic separation and mass spectrometry conditions were adapted from our previous study [37] with slight modification to obtain separation of more polar compounds. For this purpose, component A of mobile phase was enriched in water (5 mM ammonium formate in water/methanol, 2:8 *v/v*). The RPLC-Q-TOF-MS analyses (for simplicity, hereafter referred to as LC-MS analyses) were carried out in positive ionization mode only as it provided detection of all major *Synechocystis* sp. PCC 6803 cellular lipids.

Representative chromatograms obtained during the analysis of lipid extracts of *Synechocystis* sp. PCC 6803 wild type whole cells are presented in Figure S2. The database search based on measured m/z value and mass spectra interpretation revealed information about fatty acyl chain length and level of saturation of *Synechocystis* sp. PCC 6803 lipid species. It should be noted that the *sn* position of fatty acyl substituents was not evaluated. The list of identified lipids with detailed information about the mass and the retention time can be found in Table S1. Statistical tests were performed using the dataset containing only the identified lipids.

**Figure 2:**
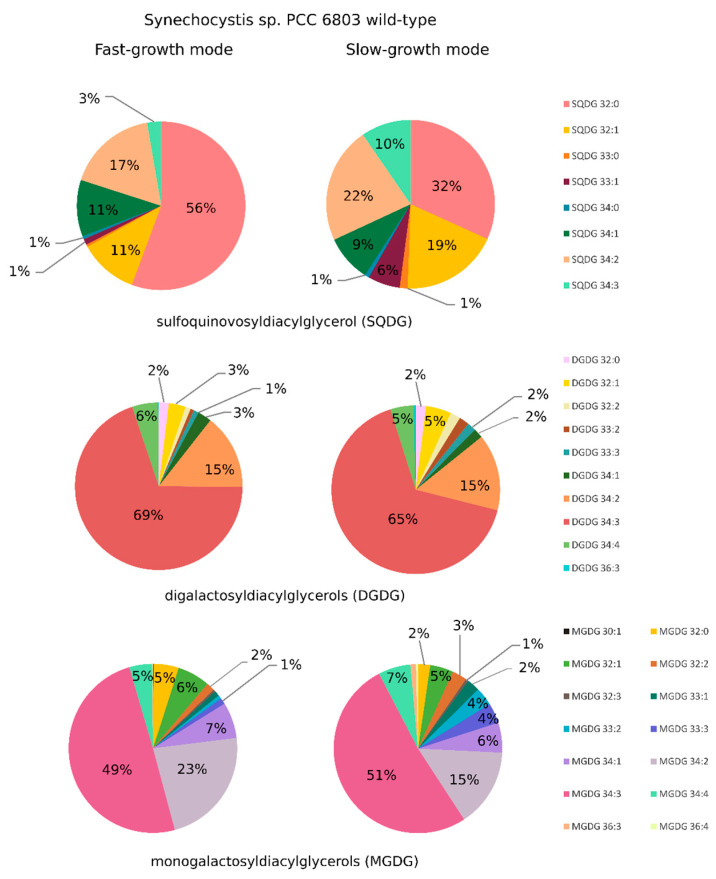
The lipidome of *Synechocystis* sp. PCC 6803 wild-type strain as determined in this work. Relative amount of lipid species (presented as average percentage) within the classes MGDG (monogalactosyldiacylglycerols), DGDG (digalactosyldiacylglycerols), and SQDG (sulfoquinovosyldiacylglycerol), detected in *Synechocystis* sp. PCC 6803 wild-type cells cultivated photoautotrophically either in fast-growth mode (FGM) or in slow-growth mode (SGM). For each lipid class, percentages were calculated based on the average peak area of each lipid species, and on the average peak area of the total lipid species within that lipid class. The detailed list of the calculated relative amounts is presented in Table S2.

The lipidome of *Synechocystis* sp. PCC 6803 wild type consisted majorly of lipid classes: monogalactosyldiacylglycerols (MGDG), digalactosyldiacylglycerols (DGDG), sulfoquinovosyldiacylglycerols (SQDG) and phosphatidylglycerols (PG). These results are in agreement with others that investigated the lipid composition of cyanobacterial cells (e.g., [13,16,38]). Within the detected classes, lipids showed a large diversity in the fatty acyl substituents length and level of unsaturation, especially among MGDG, DGDG, and SQDG (Figure 2). The most abundant species among MGDG and DGDG classes were mono- and polyunsaturated molecules containing mainly octadecatrienoic (C18:3), octadecadienoic (C18:2), and octadecenoic (C18:1) acids with hexadecanoic (C16:0) acid in their structure (e.g., DGDG 34:3 and MGDG 34:3 with fatty acid composition 18:3-16:0), in agreement with previous fatty acid composition analysis determined for *Synechocystis* sp. PCC 6803 [20,39]. As to the SQDG class, it also contained mono- and polyunsaturated species, but the most abundant lipid was SQDG 32:0 with two hexadecanoic acids in their structure (Figure 2), similarly to what was reported by [20].

In addition, four monoglycosylmonoacylglycerol species (MGMG) were also detected with C16:0, C16:1, C18:2, and C18:3 fatty acyl substituents. PG species were less diversified than other lipid classes, but similarly to MGDG and DGDG, it consisted of mono- and polyunsaturated molecules.

**Figure 3:**
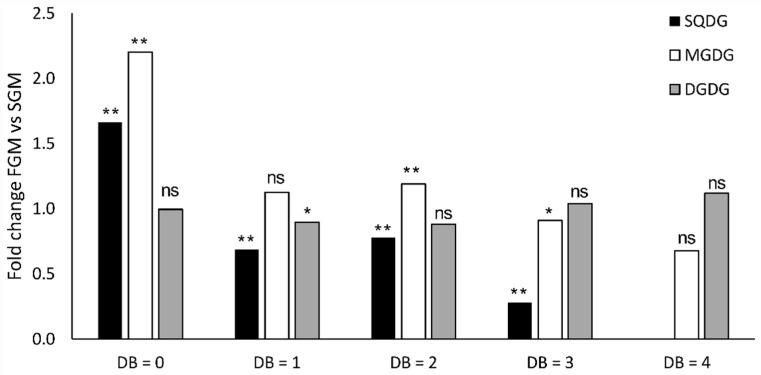
Comparison of the lipid unsaturation levels in *Synechocystis* sp. PCC 6803 wild-type strain lipidomes obtained from cells cultivated in fast- (FGM) or in slow-growth mode (SGM). For each lipid class, the indicated fold-changes were calculated based on the sum of peak areas of lipid species with particular level of unsaturation in FGM versus those in SGM. Data were normalized by the fold change of the total lipid content of each lipid class. DB, double bonds. * *p* < 0.05; ** *p* < 0.01; ns: not statistically significant difference.

**Figure 4:**
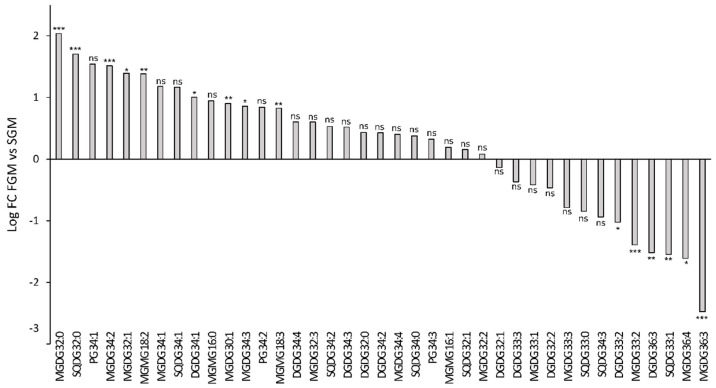
Comparison of abundance of selected lipid species detected in cells of *Synechocystis* sp. PCC 60803 cultivated in FGM and in SGM. Fold-changes were calculated by dividing the average peak area of a given lipid species detected in samples obtained in FGM (*n* = 6) by the average peak area of the same lipid species detected in samples obtained in SGM (*n* = 6). The log_2_ fold-change values are presented: positive values correspond to higher peak area in FGM compared to SGM, while negative values represent peak areas higher in SGM in comparison to FGM. Mann-Whitney unpaired analysis, * *p* < 0.05, ** *p* < 0.01, *** *p* < 0.005, ns: not statistically significant difference.

When comparing the lipidome of *Synechocystis* sp. PCC 6803 cultivated either in SGM or in FGM, several differences could be detected among the most representative lipid classes MGDG, DGDG, and SQDG (Figure 2, Figure 3 and Figure 4). In general, it was possible to observe a higher lipid content in cells cultivated in FGM as compared to cells grown in SGM: close to two-fold difference for MGDG and SQDG, while a 40% increase was observed for DGDG, even though the difference in MGDG was the only with statistical significance (see Figure S3). As cells in FGM are dividing faster, their requirement of fixed carbon is likely higher than in cells cultivated in SGM, which increases the demand for higher photosynthetic activity. As the light reactions of photosynthesis occur in the thylakoids, the most abundant membrane system in the cell, fast-dividing cells likely require more thylakoid membranes, leading to an overall increase of lipid content. Similarly, *Synechocystis* sp. PCC 6803 cells cultivated under mixotrophic conditions, which present fast growth rates, were reported to harbor a vast and more complex thylakoid membrane system than cells grown under LAHG conditions, in which the cells divide slowly [20].

Moreover, the overall level of lipid saturation was found to be higher in lipids from cells cultivated in FGM than in SGM (2-fold in MGDG lipids, and 60% in SQDG lipids, while DGDG lipids’ saturation remained approximately the same in the two growth conditions) (Figure 3). In the case of MGDG, the major constituent of cyanobacterial thylakoid membranes, and more evidently in SQDG, it is possible to observe a tendency of accumulation of saturated lipid species in FGM at the expense of polyunsaturated lipids with several double bonds per lipid, which are more abundant in SGM.

Unsaturation of fatty acids in glycerolipids can be modified by various environmental conditions, and temperature is one of the major factors determining those changes [40,41]. In higher plants and cyanobacteria, a decrease in growth temperature induces the enzymatic desaturation of fatty acids in membrane lipids. Such temperature-induced changes can be explained in terms of the regulation of membrane fluidity, which decreases at low temperatures and is restored by the enzymatic desaturation of fatty acids [42]. As temperature was maintained the same in the two cultivation setups used in this work, this environmental factor cannot account for the difference observed in unsaturation level between the FGM and the SGM lipidomes. Alterations in the extent of unsaturation of fatty acids are expected to affect the physical characteristics of the membranes and, therefore, the activities of the photosynthetic machinery [42]. It has been recognized that fatty acid desaturation is not only the primary tool for regulation of the overall membrane fluidity, but might be important through specific lipid-protein interactions, which result in structural and functional alterations [43]. In addition, thylakoid membranes of photosynthetic organisms have been shown to modify the ratio of non-bilayer/bilayer forming lipids to adapt the fluidity and the related functions to changes in the protein:lipid ratio that can occur during growth [44]. In this context, it becomes relevant to highlight that MGDG is a non-bilayer forming lipid, as it cannot be arranged in a lamellar structure due to its conical shape. However, MGDG-containing membranes can form due to the presence of membrane integral proteins, with which MGDG interacts to form stable membranous structures. Thus, the increase in fatty acid saturation levels detected in this work particularly in MGDG in cells cultivated in FGM, in line with the fact that the non-bilayer-forming propensity of MGDG depends on the level of unsaturation [43], seems to represent a specific adaptation to the physiological state in which the cells are in. Increased lipid packing in the acyl-chain region in FGM may therefore stabilize the lipid bilayer structure, which appears to be advantageous in conditions of fast growth.

For the purpose of generating biofuels, particularly biodiesel, monounsaturated fatty acids have been identified as the preferred source [45]. This is because the use of polyunsaturated fatty acids in biodiesel preparations would increase nitrogen oxide exhaust emissions, while saturated fatty acids show poor fuel properties at low temperatures, a clear drawback in cold environments [45]. From these results, FGM emerge as the most adequate growth conditions to accumulate higher lipid content, with an accumulation of saturated and monounsaturated fatty acids. However, as cyanobacteria produce a wide variety of lipid-containing fatty acids, the regulation of fatty acid composition and enrichment in particular fatty acids species may only be possible by the combination of specific growth conditions with metabolic engineering approaches [45].

Furthermore, a Mann–Whitney unpaired analysis was performed to evaluate statistically significant lipidomic differences between samples obtained from cells cultivated in FGM and in SGM. The analysis focused on identified lipids and the results are presented in Figure 4 (a more comprehensive analysis can be found in Table S3).

The detailed inspection of lipid composition shows that growth conditions significantly affect the amount of specific lipid species among all detected lipid classes. However, no clear pattern could be observed, except that lipids with shorter acyl-chains seem to be more abundant in FGM than in SGM. Lipids with shorter acyl-chain length are stiffer, therefore decreasing fluidity of the membrane. Possibly, in FGM, the combination of lipids with shorter and saturated acyl-chains represent an adaptation to key processes occurring in the thylakoid membranes, namely electron transport, regulation of light-harvesting, turnover and repair of thylakoid membrane proteins, and even membrane biogenesis, which have to be tightly controlled in fast-dividing cells. Linking the study of lipid species differentially accumulating in the two tested growth conditions (Figure 4) with the relative abundance of each lipid species within its lipid class (Figure 2), enabled to refine the analysis and to draw further conclusions. For example, one of the MGDG lipids with highest fold-change in FGM (MGDG 32:0; log_2_ fold-change of 2 (*p* < 0.005)) represents about 5% of the total MGDG lipids. Similarly, MGDG 36:3, which presents a log_2_ fold-change of −2.5 (*p* < 0.005), represents a modest 1% of the total MGDG pool in SGM. These results highlight that lipids with the highest differential accumulation are mostly among the least abundant within that specific class. The biological significance of this observation is unknown, but it may be related to specific protein:lipid interactions. Kovacs and co-workers have shown that a mere change in the oligomerization level of a membrane protein complex, resulted in altered protein-lipid interface, which affected lipid composition and, in addition, the whole dynamics of the membrane [14]. It is therefore possible that these significant changes in low abundant lipids help in stabilizing specific protein complexes and fine tuning photosynthetic-related molecular processes.

The most prominent differences in the relative levels of high-abundant lipid species were observed in the SQDG lipid class: in this case, SQDG 32:0 represents 56% of the total SQDG pool in FGM, while in SGM it represents only 32%, which corresponds to a log_2_ fold-change higher than 1.5. In addition, SQDG 32:1, SQDG 34:3 and SQDG 33:1 are relatively more abundant in SGM (19%, 10% and 6%, respectively) than in FGM (11%, 3%, and 1%, respectively). Once again, the biological meaning of these results is not entirely clear, as the biological role of the most abundant cyanobacterial lipids MGDG, DGDG, and SQDG remains to be fully understood. There are several indications in the literature that these lipids play both structural (bulk membrane lipid) and functional (specific) roles [46]. In photosynthesis, lipids have been implicated with several functions, particularly by mediating protein-protein interactions and oligomerization, by mediating protein-cofactor interactions, and by providing lipophilic regions within the protein complex [9]. For example, a high number of lipids have been detected bound within the crystal structure of photosystem II (PSII) in the cyanobacterium *Thermosynechococcus elongatus*, namely: six molecules of MGDG, four DGDG, three SQDG, and one PG per monomer, of which eleven of these lipids are located around the D1/D2 reaction center separating it from the antenna and smaller subunits [47,48]. In addition, one molecule of MGDG and two molecules of SQDG are found at the interface between two PS II monomers, thus suggesting an important structural role of these lipids for assembly and flexibility of polypeptides and cofactors within PS II [48]. In the particular case of SQDG, this is an anionic lipid whose absence in a *T. elongatus* mutant that fails to synthesize it, seriously compromised the formation of PSI trimers and PSII dimers and energy transfer in phycobilisomes [49]. These data suggest that SQDG has a specific role in the growth and photosynthesis of *T. elongatus* [49]. Moreover, it has been shown in *Synechocystis* sp. PCC 6803 that SQDG is required for activity of PSII at both donor and acceptor sides, with no crucial roles in PSI activity [50]. Therefore, the SQDG differences detected between *Synechocystis* sp. PCC 6803 cells cultivated in SGM and in FGM are likely part of a wider lipidomic adaptation program (which also includes an overall decrease in lipid unsaturation, and change in acyl-chain length, as discussed above) to adjust to the different photosynthetic activities and physiological status resulting from the alternative cultivation setups.

As briefly mentioned above, the analytical approach used in the present work enabled to detect lipid species (MGDG 33:1, MGDG 33:2, MGDG 33:3; DGDG 33:2, DGDG 33:3; SQDG 33:0, SQDG 33:1) containing odd-chain fatty acids (OCFA) belonging to the most abundant *Synechocystis* sp. PCC 6803 lipid classes (Figure 2). Identification of lipids containing OCFA has been reported in cyanobacteria (e.g., [38,51]), including in *Synechocystis* sp. PCC 6803 [13], but these lipids are extremely difficult to detect because of their low abundance [38]. Thus, the methodology employed here proofs to be robust and sensitive to detect even *Synechocystis* sp. PCC 6803 lipid species that are underrepresented in the cyanobacterial lipidome. Moreover, it was also possible to observe that cells presenting a low growth rate (cultivated in SGM) consistently contained higher content of OCFA-containing lipids than fast-dividing cells (cultivated in FGM) (Figure 2 and Figure 3). That is particularly evident when analyzing the relative abundance of these OCFA-containing lipids in each specific lipid class, representing 2.9% in FGM and 9.7% in SGM, for MGDG; 1.6% in FGM and 3.5% in SGM, for DGDG; and 1.6% in FGM and 7.7% in SGM, for SQDG. The existence of OCFA-containing lipids in other organisms than cyanobacteria has been known for long time, but their biological roles remain largely unknown. In the past, they were regarded as of low functional value [52,53], but there have been recent reports suggesting their important role, e.g., in human health [54,55]. In cyanobacteria, the knowledge in lipid biology and metabolism is lagging far behind compared to that of other organisms, and no functional role has been proposed for OCFA-containing lipids. Nevertheless, our observation that fast-dividing *Synechocystis* sp. PCC 6803 cells present lower levels of OCFA-containing lipids than cells with a slower growth rate, strongly suggest an active modulation of these lipid species accordingly to the metabolic status of the cell, which could directly affect the availability of resources and precursors. From a biosynthetic viewpoint, OCFA differ from the even-chain fatty acids. While the assembly of acetyl-CoA precursors are used for the synthesis of even-chain fatty acids, and so the carbon chain of fatty acids extends by two, resulting in even numbered chains, OCFA biosynthesis is based on a different precursor molecule, propionyl-CoA [56]. Thus, future studies will be needed to clarify not only the role of these lipids in cyanobacterial biology and physiology, but also to obtain a deeper understanding of their biosynthetic pathways.

### 2.3. Synechocystis sp. PCC 6803 tolC-Mutant Cells are Under High Oxidative Stress, Show Elevated Levels of Lipid Peroxidation, and Present Lipidome Composition Variation in response to Different Cultivation Setups

**Figure 5:**
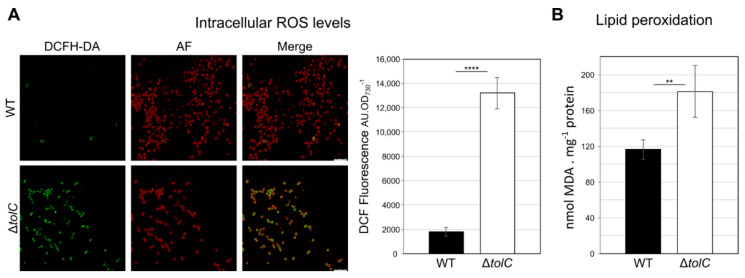
*Synechocystis* sp. PCC 6803 Δ*tolC* mutant cells present higher levels of intracellular reactive oxygen species (ROS) and lipid peroxidation than wild-type (WT) cells. (**A**) Both *Synechocystis* sp. PCC 6803 strains were incubated with the ROS detecting probe 2′,7′-dichlorodihydrofluorescein diacetate (ThermoFisher Scientific, Waltham, MA, USA), and later visualized by confocal microscopy (panels to the left). Dichlorofluorescein (DCF) signal is shown in green (DCFH-DA), while the cells’ autofluorescence (AF) is shown in red; a panel showing the result of merging the two channels is also presented. Scale bar, 10 µm. On the right-hand side, DCF fluorescence levels determined by fluorimetry are presented in arbitrary units (AU) normalized by the amount of cells (OD_730_); (**B**) Whole levels of lipid peroxidation in both cyanobacterial strains was determined by the thiobarbituric acid reactive substances assay, and it is expressed as nmol of malonaldehyde (MDA) normalized by the amount of total protein (in mg). Error bars represent standard error of the mean of at least 3 independent biological replicates. ****, *p* < 0.0001; **, *p* < 0.01.

After analyzing the lipidome of *Synechocystis* sp. PCC 6803 wild-type cells in SGM and in FGM, we decided to test the lipidomic analytical pipeline on *Synechocystis* sp. PCC 6803 mutant cells lacking a functional TolC protein (Δ*tolC* mutant, [24]). TolC is an outer membrane protein whose function is relevant in *Synechocystis* sp. PCC 6803 for the secretion of proteins [24,25,26], antibiotics [24,25], and fatty acids [27]. Two main reasons motivated the choice of the Δ*tolC* mutant for further lipidomic analyses: on one hand, the *Synechocystis* sp. PCC 6803 Δ*tolC* mutant was found to release significantly more lipid-rich, outer-membrane vesicles (or extracellular vesicles, hereafter EVs) than the wild-type strain [24]. Bacterial EVs have been described as membranous lipid particles, ranging between 20 to 500 nm in diameter, derived and released from the bacterial cell envelope and unable to duplicate [57,58,59]. Ultimately, outer-membrane vesicles can be regarded as secretion capsules of both soluble and insoluble material [58]. Hence, the hyper-vesiculation phenotype observed in the Δ*tolC* mutant strain prompted us to question whether the release of EVs to the extracellular environment could be related to specific adaptations in its lipid composition as compared to the parental strain. On the other hand, the Δ*tolC* mutant has also been shown to have its reactive oxygen species (ROS) detoxifying defenses highly upregulated and active, in particular superoxide dismutase and catalase [24]. Following up on these earlier observations, we first questioned whether the intracellular levels of ROS were higher in the mutant than in the wild-type. Using the ROS-sensitive probe DCF (dichlorodihydrofluorescein) we determined that Δ*tolC* mutant cells indeed accumulate high levels of ROS (Figure 5), and that in spite of the high activity of superoxide dismutase and catalase [24], an increase in approximately 50% could be detected in lipid peroxidation in Δ*tolC* mutant cells compared to the wild-type (Figure 5).

Remarkably, no significant changes in the growth of the mutant could be observed in relation to the wild-type, neither in SGM nor in FGM (Figure 1), in agreement with earlier observations [24]. Therefore, we hypothesized that changes in lipid composition could occur in the Δ*tolC* mutant in response to such unbalanced redox state, further supporting the need for analyzing its lipidome in detail. Hence, cells from the *Synechocystis* sp. PCC 6803 Δ*tolC* mutant strain were cultivated in SGM and in FGM, lipid extracts were obtained, and lipidomic analyses were performed as described above.

**Figure 6:**
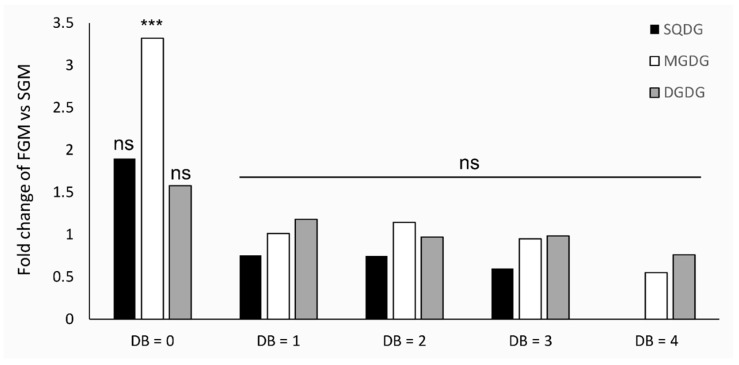
Comparison of the lipid unsaturation levels in *Synechocystis* sp. PCC 6803 *tolC*-mutant strain lipidomes obtained from cells cultivated in FGM or in SGM. For each lipid class, the indicated fold-changes were calculated based on the sum of peak areas of lipid species with particular level of unsaturation in FGM versus those in SGM. Data were normalized by the fold change of the total lipid content of each lipid class. *** *p* < 0.005; ns: not statistically significant difference.

**Figure 7:**
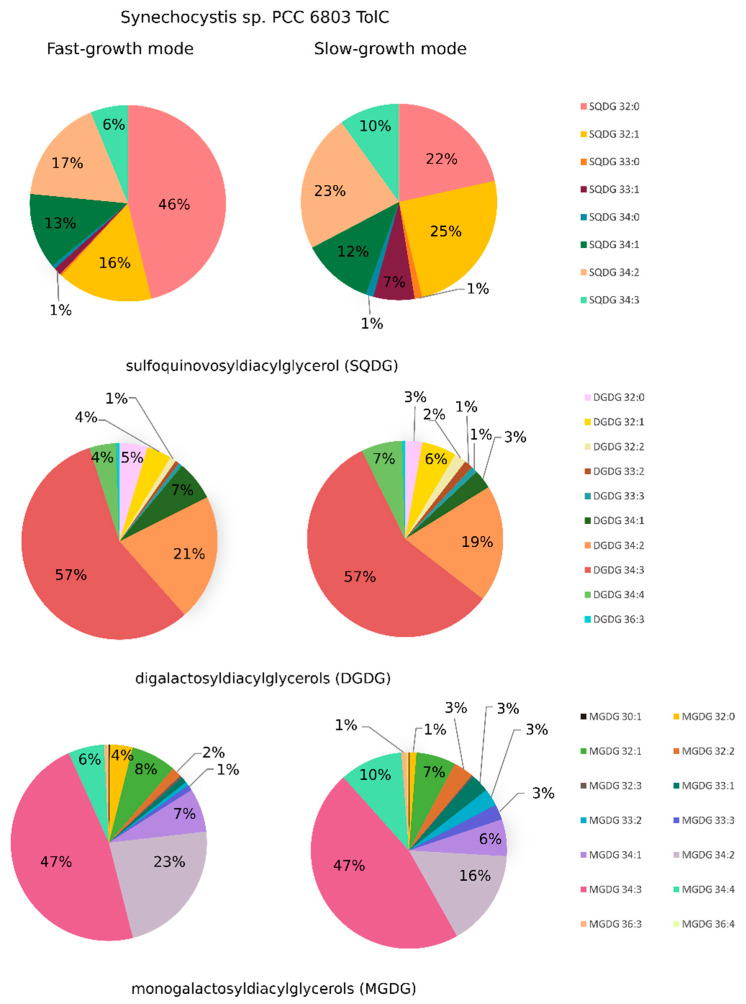
The lipidome of *Synechocystis* sp. PCC 6803 *tolC*-mutant strain as determined in this work. Relative amount of lipid species (presented as average percentage) within the classes MGDG (monogalactosyldiacylglycerols), DGDG (digalactosyldiacylglycerols), and SQDG (sulfoquinovosyldiacylglycerol), detected in *Synechocystis* sp. PCC 6803 *tolC*-mutant cells cultivated photoautotrophically either in fast-growth mode (FGM) or in slow-growth mode (SGM). For each lipid class, percentages were calculated based on the average peak area of each lipid species, and on the average peak area of the total lipid species within that lipid class.

Lipid composition analysis of the *Synechocystis* sp. PCC 6803 *tolC*-mutant revealed that this strain contains the same lipid classes as identified in the parental strain (see Table S2). However, in contrast to what was observed for the wild-type, the total lipid content of each class was found not to be statistically different between the two cultivation setups (Figure S4). In addition, the overall level of unsaturation of the detected lipids was only found to be significantly different for MGDG, with a strong accumulation of saturated lipid species (Figure 6). Further analyses were carried out, and the relative amounts of the identified lipid species within the respective lipid classes, both for cells cultivated in FGM and in cells grown in SGM are shown in Figure 7.

**Figure 8:**
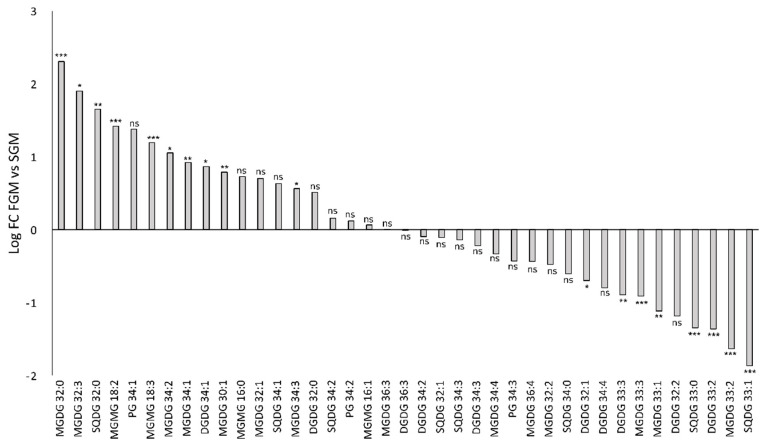
Comparison of abundance of selected lipid species detected in cells of *Synechocystis* sp. PCC 60803 *tolC*-mutant strain cultivated in FGM and in SGM. Fold-changes were calculated by dividing the average peak area of a given lipid species detected in samples obtained in FGM (*n* = 6) by the average peak area of the same lipid species detected in samples obtained in SGM (*n* = 6). The log_2_ fold-change values are presented: positive values correspond to higher peak area in FGM compared to SGM, while negative values represent peak areas higher in SGM in comparison to FGM. Mann-Whitney unpaired analysis, * *p* < 0.05, ** *p* < 0.01, *** *p* < 0.005, ns: not statistically significant difference.

Moreover, a statistical analysis was also performed on peak area of the identified lipid species between FGM and SGM conditions to evaluate significant accumulation of different lipids (Figure 8; a more comprehensive analysis can be found in Table S4).

In agreement with results obtained for the wild-type strain, no major differences could be observed in the relative content within each lipid class, except for SQDG, in which SQDG 32:0 represents 46% of the total SQDG pool in FGM while it represents 22% in SGM. That difference is accompanied with alterations in other SQDG lipid species, particularly SQDG 32:1, SQDG 34:3, and SQDG 33:1, which are more abundant in SGM than in FGM. Moreover, it was also possible to observe that lipid species with strongest differential accumulation in a particular growth condition are those that represent low abundant lipids in their respective class. For example, and similarly to what was observed for the wild-type, MGDG 32:0 was found to be more abundant in FGM (log_2_ fold-change of 2.3), but it just represents 4% of the total MGDG pool. The fact that these observations regarding modulation of the overall lipidome in cells of the *tolC*-mutant are similar to those of the parental strain, strongly support a concerted, general response of the *Synechocystis* sp. PCC 6803 lipidome in response to different growth rates, and thus to different physiological states.

### 2.4. Lipidome Composition Variation is More Evident between Cells Cultivated in SGM and in FGM than in response to the Deletion of tolC

A thorough lipidome composition comparison between samples collected from *Synechocystis* sp. PCC 6803 wild-type and *tolC*-mutant cells was also performed, in which it was included the data obtained in the two cultivation conditions tested. The goal was to identify possible specific lipidome variations related with the mutant’s phenotype, regarding its higher lipid peroxidation levels and capacity of releasing higher amounts of EVs when compared to the wild-type strain. Overall, the data indicate minor differences between the two strains, which is in line with what was presented and discussed in the previous section: irrespective of the genetic background, modulation of the lipidome composition followed the same general trends when analyzing cells cultivated in FGM or in SGM.

**Figure 9:**
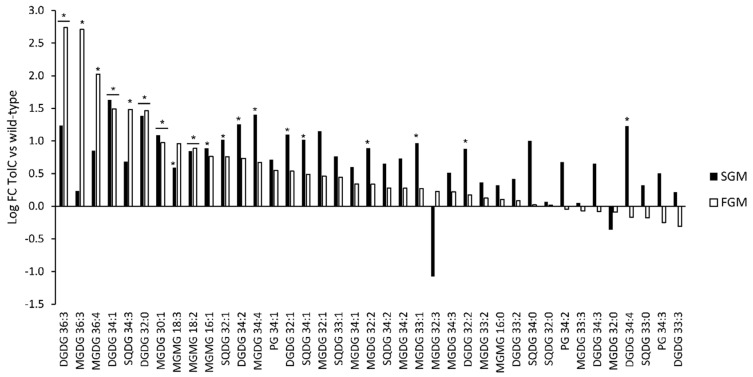
Comparison of abundance of selected lipid species detected in cells of *Synechocystis* sp. PCC 60803 wild-type and *tolC*-mutant (TolC) strains cultivated in FGM (white bars) and in SGM (black bars). Fold-changes were calculated by dividing the average peak area of a given lipid species detected in samples collected from the mutant strain in a given cultivation setup (*n* = 6) by the average peak area of the same lipid species detected in samples obtained from the wild-type in the same cultivation condition (*n* = 6). The log_2_ fold-change values are presented: positive values correspond to higher peak area in the *tolC*-mutant in comparison to wild-type, while negative values represent peak areas higher in wild-type strain in comparison to the *tolC*-mutant strain. Mann-Whitney unpaired analysis, * *p* < 0.05, bars with no asterisks—no statistically significant difference.

Nevertheless, it was possible to detect significant differential accumulation of 19 lipid species between the two strains (Figure 9; see Table S5 for details). From these 19 lipid species, five were found to accumulate differently in both cultivation setups (DGDG 32:0, DGDG 34:1, DGDG 36:3, MGDG 30:1, MGMG 18:2), while the remaining were detected in different amounts either in FGM (3 lipid species: MGDG 36:3, MGDG 36:4, SQDG34:3) or in SGM (11 lipid species: DGDG 32:1, DGDG 32:2, DGDG 34:2, DGDG 34:4, MGDG 32:2, MGDG 33:1, MGDG 34:4, MGMG 16:1, MGMG 18:3, SQDG 32:1, SQDG 34:1) only. Curiously, in spite of being a lipid class with low representation in the whole lipidome of *Synechocystis* sp. PCC 6803, three lipid species belonging to the MGMG lipid class could be identified within the group of differentially accumulating lipids. Moreover, while four of the 19 lipid species with significant differential accumulation were found to represent less than 1% of their respective lipid class, nine represent between 1% and 10%, and three represent more than 10% of their lipid classes. In the latter case, the observation is particularly relevant for DGDG 34:2, as it represents approximately 20% of the total DGDG lipid content of the *tolC*-mutant strain.

The biological interpretation of the obtained results is not easy. On one hand, the fact that no major lipidomic differences could be detected between the *tolC*-mutant and wild-type, in comparison to the differences observed in respect to the growth mode, indicates that the mutant cells do not undergo deep lipidome modulation. Although *tolC*-mutant cells are under high oxidative stress, with an accumulation of lipid peroxidation, it seems the overall lipidome is not adjusted in terms of composition. This may suggest instead a higher turnover rate of lipid metabolism in the mutant, with a possible increase in lipid biosynthesis and lipid removal and degradation, to cope with lipid damage and possible loss of lipid-dependent membrane structure and function. The accumulation of three different forms of the lysolipid class MGMG (MGMG 16:1, 18:2 and 18:3) in the mutant could be an indication supporting such scenario. Further work needs to be carried out to elucidate this possibility. On the other hand, and at a first glance, the high vesiculation capacity showed by the mutant does not seem either to induce lipidome composition adaptations. However, the 19 lipid species that were detected to accumulate differently between the wild-type and mutant indicate that certain specific changes are indeed required, especially those five lipids that accumulate more in the mutant than in the wild-type in both cultivation conditions. As *Synechocystis* sp. PCC 6803 is an organism with three different membrane systems, each fulfilling a different cellular and physiological role, it is likely that adjustments in lipid composition occur in one particular membrane, but not in all. In the case of EVs formation, it is possible that the outer membrane represents the key membrane system that requires lipid composition variation to modulate the EVs production capacity. Thus, future studies will have to focus on the lipid composition dynamics of each membrane system separately, which will require the isolation of those membranes. Finally, analysis of the lipid composition of cyanobacterial EVs also represents a point of interest, as the lipid composition of these nanostructures remains largely unknown. Validation of the present untargeted lipidomics platform for cyanobacterial lipids opens the door to more detailed and focused studies.

## 3. Materials and Methods

### 3.1. Strains and Growth Conditions

The unicellular cyanobacterium *Synechocystis* sp. PCC 6803 wild-type (non-motile and with S-layer) and respective *tolC*-deletion mutant [24] were maintained in 100 mL Erlenmeyer flasks, containing liquid BG11 medium [60] and supplemented with kanamycin at a final concentration of 100 µg mL^−1^ in the case of the *tolC*-mutant, at 30 °C, under a 12 h light (25 µmol photons m^−2^ s^−1^)/12 h dark regime. For lipid analysis, a seed culture kept under maintenance conditions, with an optical density (730 nm) of approximately 2–3 was used to prepare the “inoculum culture”. Thus, a single culture of 200 mL was set up with an initial OD_730_ of 0.05 from the seed culture, and cultivated in glass gas-washing bottles, with aeration (1 L air min^−1^), at 27 °C, under a 16 h light (40 µmol photons m^−2^ s^−1^)/8 h dark regime. When the “inoculum culture” reached an OD_730_ between 1.0 and 1.5, cells from this culture were used for two different cultivation setups: in fast-growth conditions (FGM), cultures were inoculated at an optical density (730 nm) of 0.05, and grown in BG11 medium (culture total volume of 200 mL) in glass gas-washing bottles, with aeration (1 L air min^−1^), at 27 °C, under a 16 h light (40 µmol photons m^−2^ s^−1^)/8 h dark regime for 4 days, resulting in a final OD_730_ of approximately 1.4. In slow-growth conditions (SGM), cultures were inoculated at an OD_730_ of 0.1, and grown in BG11 medium (culture total volume of 250 mL) in Erlenmeyer flasks, with orbital shaking (approximately 90 r.p.m.), at 27 °C, under a 16 h light (40 µmol photons m^−2^ s^−1^)/8 h dark regime also for 4 days, resulting in a final OD_730_ of approximately 0.5.

### 3.2. Sample Preparation

After cultivation, cells were collected by centrifugation (4000× *g*) at room temperature, and pellets were washed with BG11 medium. Cells were then snap-frozen in liquid nitrogen, saved at −80 °C, and later subjected to lyophilization. Freeze-dried cells were saved at room temperature until further analysis. For obtaining whole cell extracts for lipid analysis, the method described by Bligh and Dyer [61] was used, with modifications. In brief, 12 mg of freeze-dried cyanobacterial biomass were suspended in 500 µL Milli-Q grade water. Next, 450 µL of cell suspension were mixed with 1.9 mL of chloroform/methanol mixture (1:2, *v/v*) and with 0.6 g of acid-washed glass beads in a glass vial, and vortexed vigorously for 7 min. Later, 625 µL of chloroform were added to the mixture, and vortexed for 10 s; 625 µL of Milli-Q grade water were also added, and the mixture vortexed for 60 s, before being separated by centrifugation (4000× *g*, at room temperature). The organic phase was collected to a new glass tube, while the mixture was subjected to 3 additional rounds of extraction with chloroform. The organic phase obtained with the various extractions was combined in a single tube, subjected to centrifugation and the contaminating aqueous phase discarded. Samples were dried with a gentle stream of nitrogen, and saved at room temperature until further analysis. Dried lipid extracts were dissolved in 300 µL of chloroform and 10 µL of the dissolved extract was transferred to a glass insert with 140 µL of mobile phase component A (5 mM ammonium formate in water/methanol, 2:8 *v/v*). Subsequently, extracts were analysed by reversed-phase liquid chromatography–quadrupole-time of flight mass spectrometry (RPLC-Q-TOF-MS).

### 3.3. Reversed-Phase Liquid Chromatography–Quadrupole-Time of Flight Mass Spectrometry (RPLC-Q-TOF-MS) and Tandem Mass Spectrometry (MS/MS) Analysis of Lipid Extracts

Analysis was performed on an Agilent 1290 LC system equipped with a binary pump, an online degasser, an autosampler and a thermostated column compartment coupled with a 6540 quadrupole-time of flight mass spectrometer with a Dual ESI source (Agilent Technologies, Santa Clara, CA, USA). Agilent ZORBAX SB-C-18 50 × 2.1 mm, 1.8 μm column (Agilent Technologies, Santa Clara, CA, USA) with in-line filter 0.2 μm (Agilent Technologies, Santa Clara, CA, USA) was used for crude lipid extract separation. The RPLC-Q-TOF-MS method (for simplicity, hereafter referred to as LC-MS) was adapted with slight modification from previous studies [37]. The mobile phase consisted of component A: 5 mM ammonium formate in water/methanol (2:8 *v/v*) and component B: 5 mM ammonium formate in water/n-hexane/2-propanol (1:20:79 *v/v/v*). Gradient elution program was 0–60% B at 0–20 min, 60–100% B at 20–22 min, and maintaining for 2 min. Then, after 0.5 min the eluent was returned to 0% B, followed by 10 min equilibration with this mobile phase composition prior to the next injection. The column temperature throughout analyses was 45 °C. The flow rate of mobile phase was 0.3 mL min^−1^. Injection volume was set at 1 μL. During the analysis samples were kept in autosampler at 4 °C.

Electrospray ionization (ESI) source was operated in positive ion mode with the conditions as follows: fragmentor voltage at 120 V, nebulizer gas at 35 psig, capillary voltage at 3500 V, drying gas flow rate and temperature at 10 L min^−1^ and 300 °C, respectively. Data were acquired in centroid and profile mode using the High Resolution mode (4 GHz). The mass range were set at 100–1700 in MS mode. The time of flight (TOF) was calibrated on the daily basis, before sample analysis and also using reference masses at m/z 121.050873, 149.02332, 922.009798, 1221.990637 during the analysis run to allow constant mass correction. Quality control (QC) samples were analyzed at the beginning and at the end of the batch and every 4 injections throughout the run to provide a measurement of the stability and performance of the LC-MS system. Extraction blank sample was prepared in the same way as biological test samples (extraction procedure with the use of deionized water instead of bacterial cells) and used to monitor and eliminate compounds that were not bacterial cellular lipids, but originated from chemicals used for extraction or LC-MS analysis.

Tandem mass spectrometry experiments were carried out to evaluate fatty acyl substituents of identified lipids. Chromatographic conditions and ion source parameters were identical to those during LC-MS analysis. The two most abundant precursor ions were selected for fragmentation with the collision energy 35 V and excluded for 0.3 min. The monitored m/z range was 50–1700 amu.

### 3.4. Data Treatment and Analysis

The LC-MS raw data were processed with the MassHunter Workstation Profinder Software, version B.10.0 (Agilent Technologies, Santa Clara, CA, USA). The peak areas of the identified lipids (a list of these lipids is presented in Table S1) were obtained using the Batch Targeted Feature Extraction algorithm with the following parameters: positive ions, charge carriers—H+, Na+, NH4+; match tolerance, 15 ppm; retention time, 0.3 min; Gaussian smoothing before extracted ion chromatogram extraction (EIC) filtering on peak height, 1000 counts. In both data pre-treatment approaches, the .cef files were exported and imported to Mass Profiler Professional 15.1 software (Agilent Technologies, Santa Clara, CA, USA) for data alignment and filtration. Missing values were exported as missing. The alignment parameters were set as follows: alignment slope = 0.0%; alignment intercept = 0.4 min; mass tolerance slope = 20.0 ppm; intercept = 2.0 mDa. Filtration was based on frequency (the molecular features (MFs) remained in the dataset if they were present in 100% of the samples in at least one specified group) and the QC% of the relative standard deviation (%RSD) (MFs remained if %RSD < 20% in all the QC samples). The MFs that were present in the extraction blank, with the average peak volume higher than 10% of the average peak volume in the real samples, were removed. The statistical analyses and fold change calculation (Mann–Whitney unpaired test) were conducted using Mass Profiler Professional 15.1 software (Agilent Technologies, Santa Clara, CA, USA). Statistical tests and fold change (FC) calculations were conducted using the average peak area of samples within defined group of samples. The percentage relative amount of lipids within the specified lipid class was calculated in Microsoft Excel 2016 software (Microsoft Corporation, Redmond, WA, USA) by dividing the lipid species peak area by the sum of the peak area of all lipid species detected within the class.

Principal component analysis (PCA) and partial last square-discriminant analysis (PLS-DA) were performed with the help of Metabolanalyst on-line service (https://www.metaboanalyst.ca/) [62]. Data was autoscaled and transformed logarithmically. The PCA was used to visualize the data and check LC-MS stability. The QC sample clustering on the score plot demonstrated the stability and repeatability of the analytical system used.

### 3.5. Lipid Group Identification

Lipids statistically significant for strains differentiation were tentatively identified by searching the lipid database created in MassHunter Personal Databse & Library, version B.03.01, build 86.0 (Agilent Technologies, Santa Clara, CA, USA) [37]. Created database contains 18 lipid main classes, 36 lipid subclasses (named and classified accordingly to Lipid Maps, www.lipidmaps.org) and 7047 lipid groups (accordingly to [63]). Theoretical lipids contained in the database are in accordance with the literature regarding *Synechocystis* sp. PCC 6803 lipids (e.g., [13,16,20]). Automatic database search was performed by MassHunter Qualitative Software with the following parameters: values to match, mass only; match tolerance, 15 ppm; charge carriers, H+, Na+, NH4+, K+; charge state: 1–2. Interpretation of MS/MS spectra was performed to confirm lipid class and fatty acyl substituents.

### 3.6. Determination of Intracellular Reactive Oxygen Species (ROS) and Lipid Peroxidation Levels

To determine the intracellular levels of reactive oxygen species in *Synechocystis* sp. PCC 6803 wild-type and *tolC*-mutant strains, cells were cultivated in FGM up to an OD_730_ of approximately 1.5. Ten mL of each culture were centrifuged for 10 min at 4000× *g*, and cells were suspended in BG11 medium to an OD_730_ of 10. Serial dilutions were prepared in a final volume of 1 mL, to final OD_730_ of 2, 1 and 0.5. Twenty µL of 0.5 mM 2′,7′-dichlorodihydrofluorescein diacetate (H_2_DCFDA) probe (ThermoFisher Scientific, Waltham, MA, USA) were added to the cells, before being incubated in darkness at 30 °C for 1 h. After the incubation period, cells were collected by centrifugation at 5000× *g* for 8 min and suspended in 1 mL of fresh BG11 medium. 200 µL of each suspension were placed on a 96-well plate and 2′,7′-dichlorofluorescein (DCF) fluorescence was detected using a Synergy Mx plate reader (BioTek, Winooski, VT, USA). ROS levels are expressed as arbitrary DCF fluorescent units and normalized per OD_790_ (AU OD^−1^). After determining DCF fluorescence levels, cells used in the assay were visualized using a Leica SP2 AOBS SE laser scanning confocal microscope. Cells were loaded on 1% low-melting-point agarose beds dissolved in BG11, and covered with a clover slip. DCF emission was collected between 495 and 560 nm, and cyanobacterial autofluorescence was acquired between 620 and 670 nm, when cells were exposed to an Ar laser beam of 488 nm. Wild-type cells not incubated with H_2_DCFDA were used to define the basal autofluorescence signal in the DCF channel, and the same acquisition settings were used throughout the various experiments.

To evaluate the level of lipid peroxidation, both wild-type and *tolC*-mutant strains were investigated using the thiobarbituric acid reactive substances assay. Fifty mL of culture of each strain cultivated up to an OD_730_ of 1 in FGM were centrifuged at 4000× *g* for 10 min. Cells were washed in 1 mL of 20 mM sodium phosphate buffer pH 7.0 and collected by centrifugation at 16,100× *g* for 2 min. The cell pellet was suspended in 250 µL of 20 mM sodium phosphate buffer pH 7.0 and disrupted by sonication (Branson sonifier, Brookfield, CT, USA, operated in 4 cycles of 15 s each, under an output of 3 and duty cycle of 50%). Then, 50 µL of sample was taken for protein determination using the Bradford method, and 28 µL of 100% (*w/v*) trichloroacetic acid (TCA) were added to the remaining lysate. The mixture was vigorously vortexed in 2 cycles of 1 min each, with 1 min interval resting on ice. This mixture was centrifuged at 10,000× *g* for 10 min at 4 °C and the supernatant was used for subsequent steps. 100 µL of the extract were well mixed with 100 µL of 0.1 M EDTA pH 8.0, 600 µL of freshly prepared TBA solution (15% (*w/v*) TCA, 250 mM HCl, and 26 mM 2-thiobarbituric acid) and 8.1 µL of freshly prepared 50 mM butylated hydroxytoluene dissolved in ethanol. The reaction mixture was incubated at 100 °C for 15 min, cooled at room temperature for 10 min and on ice for an additional 5 min, after which absorbance at 532 nm was measured. Lipid peroxidation is expressed as the amount of malonaldehyde (MDA) present in the reaction mixture (nmol MDA, using the extinction coefficient determined by [64] and normalized by amount of protein.

## 4. Conclusions

Cyanobacteria have an enormous potential of becoming next-generation microbial cell factories. For this reason, a series of analytical solutions must be available to robustly evaluate alterations in the composition of various types of biomolecules in response to metabolic engineering and/or optimized cultivation conditions. Here, we present and validate an untargeted lipidomics approach to analyze the lipid content of the model cyanobacterium *Synechocystis* sp. PCC 6803. We demonstrate that the established pipeline allows for the sensitive detection and detailed analysis of lipid composition variation, with cells grown in alternative cultivation setups, and with a genetically modified strain. Curiously, in spite of being cultivated under photoautotrophic conditions, the two tested setups resulted in significantly different growth-rates, which were followed by strong overall lipid composition adaptations (total lipid content and unsaturation, relative differences within the SQDG lipid class, differential accumulation of specific lipid species). However, lipid species with higher differential accumulation between the cultivation setups were among those with lowest relative amount within its lipid class, and the biological relevance of these findings is not obvious. This fact further highlights that much remains to be explored in terms of lipid composition modulation in cyanobacteria in response to different environmental cues. The *tolC*-mutant strain also showed lipid composition changes when cultivated either in FGM or in SGM, similar to those observed for the wild-type strain. Nevertheless, significant differences between the mutant and the parental strain were restricted to 19 lipid species that were found to accumulate differentially. As cyanobacteria have different membrane systems (thylakoids, plasma membrane, and outer membrane), each with a particular lipid composition, lipidomic studies in response to cultivation conditions and genetic modifications will benefit, in future studies, from the isolation of specific membranes. The present work focused on the modulation of the total pools of lipids extracted from whole cells, but alterations in the lipid composition in specific membranes may shed light on alterations that may be underrepresented in the total content of cellular lipids. The identification of such alterations may contribute towards a better understanding of particular physiological responses in cyanobacteria, which will help in clarifying basic biological processes, as well as develop these photosynthetic prokaryotes to become robust and sustainable production platforms.

## Supplementary Materials

The following are available online at https://www.mdpi.com/1422-0067/21/23/8883/s1.

## Abbreviations

DG	diacylglycerol
MGDG	monogalactosyldiacylglycerol
DGDG	digalactosyldiacylglycerol
SQDG	sulfoquinovosyldiacylglycerol
PG	Phosphatidylglycerol
LAHG	Light-activated heterotrophic growth
FGM	Fast-growth mode
SGM	Slow-growth mode
PS	Photosystem
OCFA	Odd-chain fatty acids
EVs	Extracellular vesicles
DCF	Dichlorodihydrofluorescein
ROS	Reactive oxygen species
