# Supplementary material for: Untargeted Lipidomics Analysis of the Cyanobacterium Synechocystis sp. PCC 6803: Lipid Composition Variation in Response to Alternative Cultivation Setups and to Gene Deletion
Source: Int J Mol Sci. 2020 Nov 24;21(23):8883. doi: 10.3390/ijms21238883 (PMC7727718; doi:10.3390/ijms21238883)
Supplement: Supplementary file 1 [file ijms-21-08883-s001.pdf]

International Journal of
Molecular Sciences

MDPI

Supplementary Materials

Untargeted Lipidomics Analysis of the Cyanobacterium *Synechocystis* sp. PCC 6803: Lipid Composition Variation in Response to Alternative Cultivation Setups and to Gene Deletion

Weronika Hewelt-Belka [1], Ágata Kot-Wasik [1], Paula Tamagnini [2,3,4] and Paulo Oliveira [2,3,*]

1 Department of Analytical Chemistry, Faculty of Chemistry, Gdańsk University of Technology, Gabriela Narutowicza 11/12, 80-233 Gdańsk, Poland; werbelka@pg.edu.pl (W.H.-B.); agawasik@pg.edu.pl (Á.K.-W.)
2 i3S—Instituto de Investigação e Inovação em Saúde, Universidade do Porto, R. Alfredo Allen 208, 4200-135 Porto, Portugal; pmtamagn@ibmc.up.pt
3 IBMC—Instituto de Biologia Molecular e Celular, Universidade do Porto, R. Alfredo Allen 208, 4200-135 Porto, Portugal
4 Departamento de Biologia, Faculdade de Ciências, Universidade do Porto, R. Campo Alegre s/n, 4169-007 Porto, Portugal
* Correspondence: paulo.oliveira@ibmc.up.pt; Tel.: +351-22-607-4900

Received: 30 October 2020; Accepted: 21 November 2020; Published: date

Contents list

Table S1. List of identified lipid compounds in *Synechocystis* sp. PCC 6803.

Table S2. Relative amount of lipid species within the classes MGDG, DGDG, and SQDG detected in *Synechocystis* sp. PCC 6803 wild-type and *tolC*-mutant strain cells cultivated photoautotrophically either in FGM or in SGM.

Table S3. Comparison of abundance of specific lipid species detected in cells of *Synechocystis* sp. PCC 60803 cultivated in FGM and in SGM.

Table S4. Comparison of abundance of specific lipid species detected in cells of Synechocystis sp. PCC 60803 tolC-mutant strain cultivated in FGM and in SGM.

Table S5. Comparison of lipid composition between TolC and WT in FGM and SGM.

Figure S1. Analytical workflow used for the lipidomic analysis of *Synechocystis* sp. PCC 6803 cells.

Figure S2. Representative ion chromatogram of the most abundant lipid species of *Synechocystis* sp. PCC 6803 wild-type.

Figure S3. Total lipid content among the MGDG, DGDG and SQDG classes of the *Synechocystis* sp. PCC 6803 wild-type strain.

Figure S4. Total lipid content among the MGDG, DGDG and SQDG classes of the *Synechocystis* sp. PCC 6803 *tolC*-mutant strain.

Table S1. List of identified lipid compounds in *Synechocystis* sp. PCC 6803. When possible, fatty acyl composition was evaluated by the MS/MS experiments.

Lipid name	Fatty acyl composition	Formula	Neutral Mass	Retention Time [min]
DGDG 32:0	16:0-16:0	C47 H88 O15	892.6135	10.6
DGDG 32:1	16:0-16:1	C47 H86 O15	890.5976	9.8
DGDG 32:2		C47 H84 O15	888.5813	9.2
DGDG 33:2		C48 H86 O15	902.5971	9.7
DGDG 33:3		C48 H84 O15	900.5813	9.1
DGDG 34:1	16:0-18:1	C49 H90 O15	918.6283	10.9
DGDG 34:2	16:0-18:2	C49 H88 O15	916.6130	10.2
DGDG 34:3	16:0-18:3	C49 H86 O15	914.5983	9.6
DGDG 34:4	16:1-18:3	C49 H84 O15	912.5817	8.9
DGDG 36:3		C51 H90 O15	942.6283	10.6
MGDG 30:1		C39 H72 O10	700.5107	9.8
MGDG 32:0	16:0-16:0	C41 H78 O10	730.5603	11.8
MGDG 32:1	16:0-16:1	C41 H76 O10	728.5447	10.9
MGDG 32:2	16:0-16:2	C41 H74 O10	726.5310	10.2
MGDG 32:3	18:3-14:0	C41 H72 O10	724.5128	9.5
MGDG 33:1	16:0-17:3	C42 H78 O10	742.5606	11.5
MGDG 33:2		C42 H76 O10	740.5442	10.7
MGDG 33:3		C42 H74 O10	738.5289	10.1
MGDG 34:1	16:0-18:1	C43 H80 O10	756.5759	12.0
MGDG 34:2	16:0-18:2	C43 H78 O10	754.5607	11.3
MGDG 34:3	16:0-18:3	C43 H76 O10	752.5464	10.7
MGDG 34:4	16:1-18:3	C43 H74 O10	750.5297	10.0
MGDG 36:3	16:0-20:3	C45 H80 O10	780.5757	11.7
MGDG 36:4		C45 H78 O10	778.5600	11.0
MGMG 16:0		C25 H48 O9	492.3299	3.1
MGMG 16:1		C25 H46 O9	490.3145	2.2
MGMG 18:3		C27 H46 O9	514.3139	2.1
MGMG18:2		C27 H48 O9	516.3301	2.7
PG 34:1		C40 H77 O10 P	748.5248	8.8
PG 34:2	16:0-18:2	C40 H75 O10 P	746.5117	8.1
PG 34:3	16:0-18:3	C40 H73 O10 P	744.4946	7.5
SQDG 32:0	16:0-16:0	C41 H78 O12 S	794.5225	7.8
SQDG 32:1	16:1-16:0	C41 H76 O12 S	792.5068	7.1
SQDG 33:0		C42 H80 O12 S	808.5382	8.3
SQDG 33:1		C42 H78 O12 S	806.5225	7.6
SQDG 34:0		C43 H82 O12 S	822.5536	8.8

SQDG 34:1	16:0-18:1	C43 H80 O12 S	820.5379	8.1
SQDG 34:2	16:0-18:2	C43 H78 O12 S	818.5228	7.5
SQDG 34:3	16:0-18:3	C43 H76 O12 S	816.5069	7.0

Table S2. Relative amount of lipid species (presented as average percentage) within the classes MGDG (monogalactosyldiacylglycerols), DGDG (digalactosyldiacylglycerols), and SQDG (sulfoquinovosyldiacylglycerol) detected in *Synechocystis* sp. PCC 6803 wild-type and *tolC*-mutant strain cells cultivated photoautotrophically either in fast-growth mode (FGM) or in slow-growth mode (SGM). For each lipid class, percentages were calculated based on the average peak area of each lipid species, and on the average peak area of the total lipid species within that lipid class.

	Wild-type				*tolC*-mutant			
	FGM average % relative amount (n=6)	FGM standard deviation	SGM average % relative amount average (n=6)	SGM standard deviation	FGM average % relative amount (n=6)	FGM standard deviation	SGM average % relative amount average (n=6)	SGM standard deviation
				**MGDG**				
MGDG 30:1	0.099%	0.019%	0.100%	0.019%	0.159%	0.023%	0.135%	0.023%
MGDG 32:0	4.82%	0.85%	2.23%	0.42%	3.74%	0.64%	1.10%	0.12%
MGDG 32:1	6.5%	2.3%	4.7%	1.6%	7.5%	2.5%	6.6%	1.7%
MGDG 32:2	1.56%	0.12%	2.81%	0.38%	1.64%	0.25%	3.31%	0.24%
MGDG 32:3	0.453%	0.051%	0.59%	0.17%	0.437%	0.040%	0.29%	0.28%
MGDG 33:1	0.98%	0.25%	2.44%	0.36%	0.96%	0.10%	3.06%	0.48%
MGDG 33:2	0.701%	0.077%	3.46%	0.18%	0.64%	0.11%	2.87%	0.34%
MGDG 33:3	1.18%	0.18%	3.84%	0.30%	0.92%	0.06%	2.54%	0.23%
MGDG 34:1	7.3%	2.9%	5.8%	1.2%	7.2%	1.4%	6.1%	2.9%
MGDG 34:2	22.5%	2.2%	14.83%	0.47%	22.9%	5.4%	15.9%	2.4%
MGDG 34:3	49.0%	4.2%	51.0%	3.7%	47.2%	5.4%	46.5%	1.6%
MGDG 34:4	4.8%	2.2%	6.7%	2.8%	5.9%	1.7%	10.40%	0.81%
MGDG 36:3	0.106%	0.034%	1.08%	0.30%	0.544%	0.055%	0.793%	0.068%
MGDG 36:4	0.075%	0.040%	0.38%	0.11%	0.218%	0.029%	0.431%	0.061%
				**SQDG**				
SQDG 32:0	55.5%	2.1%	31.7%	2.1%	46.13%	0.94%	21.54%	0.48%
SQDG 32:1	11.3%	1.4%	18.8%	1.1%	15.7%	2.0%	24.63%	0.90%
SQDG 33:0	0.453%	0.083%	1.50%	0.18%	0.333%	0.086%	1.25%	0.31%
SQDG 33:1	1.15%	0.16%	6.17%	0.47%	1.268%	0.068%	6.79%	0.48%
SQDG 34:0	0.71%	0.52%	1.06%	0.64%	0.63%	0.52%	1.29%	0.60%
SQDG 34:1	10.9%	1.0%	8.98%	0.78%	12.5%	1.0%	11.79%	0.38%
SQDG 34:2	17.30%	0.64%	22.25%	0.70%	17.3%	1.2%	22.71%	0.76%
SQDG 34:3	2.70%	0.25%	9.59%	0.93%	6.19%	0.50%	9.99%	0.67%
				**DGDG**				
DGDG32:0	1.94%	0.24%	1.94%	0.15%	4.63%	0.50%	2.83%	0.33%

DGDG32:1	3.22%	0.16%	4.79%	0.11%	4.06%	0.45%	5.70%	0.36%
DGDG32:2	1.041%	0.061%	1.96%	0.22%	1.02%	0.13%	2.00%	0.14%
DGDG33:2	0.695%	0.031%	1.92%	0.11%	0.640%	0.059%	1.43%	0.17%
DGDG33:3	0.940%	0.058%	1.65%	0.15%	0.659%	0.057%	1.06%	0.07%
DGDG34:1	2.70%	0.26%	1.8%	0.3%	6.57%	0.48%	3.15%	0.45%
DGDG34:2	14.6%	2.1%	14.6%	1.5%	20.8%	1.4%	19.3%	1.2%
DGDG34:3	69.3%	2.4%	65.5%	3.1%	56.6%	1.5%	57.3%	2.2%
DGDG34:4	5.5%	2.1%	5.5%	3.6%	4.4%	2.4%	6.7%	3.7%
DGDG36:3	0.091%	0.021%	0.348%	0.052%	0.54%	0.16%	0.46%	0.11%

Table S3. The comparison of abundance of specific lipid species detected in cells of *Synechocystis* sp. PCC 60803 wild-type (WT) strain cultivated in FGM and in SGM. Fold-changes were calculated by dividing the average peak area of a given lipid species detected in samples obtained in FGM (n=6) by the average peak area of the same lipid species detected in samples obtained in SGM (n=6) (Mann-Whitney unpaired analysis, $p < 0.05$).

Lipid	p WT FGM vs WT SGM	Fold change (FC) WT FGM vs WT SGM	Log FC (WT FGM vs WT SGM)
MGDG 32:0	0.004	4.3	2.0
SQDG 32:0	0.004	3.7	1.7
PG 34:1	0.2	3.3	1.5
MGDG 34:2	0.004	2.8	1.5
MGDG 32:1	0.03	2.6	1.4
MGMG 18:2	0.006	3.1	1.4
MGDG 34:1	0.06	2.5	1.2
SQDG 34:1	0.06	2.4	1.2
DGDG 34:1	0.04	2.2	1.0
MGMG 16:0	0.1	2.1	0.9
MGDG 30:1	0.01	1.9	0.9
MGDG 34:3	0.03	1.8	0.9
PG 34:2	0.3	1.9	0.8
MGMG 18:3	0.02	2.0	0.8
DGDG 34:4	0.1	1.6	0.6
MGDG 32:3	0.08	1.5	0.6
SQDG 34:2	0.3	1.5	0.5
DGDG 34:3	0.3	1.5	0.5
DGDG 32:0	0.4	1.4	0.4
DGDG 34:2	0.5	1.4	0.4
MGDG 34:4	0.6	1.2	0.4
SQDG 34:0	1.0	1.0	0.4
PG 34:3	0.6	1.4	0.3
MGMG 16:1	0.9	1.3	0.2
SQDG 32:1	0.8	1.2	0.2

MGDG 32:2	0.6	1.1	0.1
DGDG 32:1	0.6	1.0	-0.1
DGDG 33:3	0.3	0.8	-0.4
MGDG 33:1	0.2	0.8	-0.4
DGDG 32:2	0.2	0.8	-0.5
MGDG 33:3	0.06	0.6	-0.8
SQDG 33:0	0.06	0.6	-0.8
SQDG 34:3	0.06	0.6	-0.9
DGDG 33:2	0.04	0.5	-1.0
MGDG 33:2	0.004	0.4	-1.4
DGDG 36:3	0.01	0.4	-1.5
SQDG 33:1	0.01	0.3	-1.5
MGDG 36:4	0.04	0.4	-1.6
MGDG 36:3	0.004	0.2	-2.5

Table S4. Comparison of abundance of specific lipid species detected in cells of *Synechocystis* sp. PCC 60803 *tolC*-mutant strain cultivated in FGM and in SGM. Fold-changes were calculated by dividing the average peak area of a given lipid species detected in samples obtained in FGM (n=6) by the average peak area of the same lipid species detected in samples obtained in SGM (n=6) (Mann-Whitney unpaired analysis, $p < 0.05$).

Lipid	p tolC FGM vs tolC SGM	Fold change (FC) tolC FGM vs tolC SGM	Log FC (tolC FGM vs tolC SGM]
MGDG 32:0	0.004	4.8	2.3
MGDG 32:3	0.03	2.3	1.9
SQDG 32:0	0.01	3.0	1.7
MGMG 18:2	0.004	2.7	1.4
PG 34:1	0.06	2.0	1.4
MGMG 18:3	0.004	2.3	1.2
MGDG 34:2	0.04	2.1	1.1
MGDG 34:1	0.01	1.8	0.9
DGDG 34:1	0.04	1.7	0.9
MGDG 30:1	0.02	1.7	0.8
MGMG 16:0	0.06	1.7	0.7
MGDG 32:1	0.1	1.6	0.7
SQDG 34:1	0.08	1.5	0.6
MGDG 34:3	0.04	1.4	0.6
DGDG 32:0	0.2	1.4	0.5
SQDG 34:2	0.6	1.1	0.2
PG 34:2	0.6	1.0	0.1
MGMG 16:1	0.9	1.0	0.1

MGDG 36:3	1.0	1.0	0.0
DGDG 36:3	0.8	1.0	0.0
DGDG 34:2	0.8	0.9	-0.1
SQDG 32:1	0.8	0.9	-0.1
SQDG 34:3	0.6	0.9	-0.1
DGDG 34:3	0.4	0.9	-0.2
MGDG 34:4	0.3	0.8	-0.3
PG 34:3	0.2	0.7	-0.4
MGDG 36:4	0.1	0.7	-0.4
MGDG 32:2	0.2	0.7	-0.5
SQDG 34:0	0.2	0.7	-0.6
DGDG 32:1	0.04	0.6	-0.7
DGDG 34:4	0.2	0.7	-0.8
DGDG 33:3	0.01	0.5	-0.9
MGDG 33:3	0.004	0.5	-0.9
MGDG 33:1	0.006	0.4	-1.1
DGDG 32:2	0.004	0.4	-1.2
SQDG 33:0	0.004	0.4	-1.3
DGDG 33:2	0.004	0.4	-1.4
MGDG 33:2	0.004	0.3	-1.6
SQDG 33:1	0.004	0.3	-1.9

Table S5. The comparison of lipid composition between *Synechocystis* sp. PCC 60803 *tolC*-mutant and wild-type (WT) strains in FGM and SGM (Mann-Whitney unpaired analysis, $p < 0.05$). Fold-changes were calculated by dividing the average peak area of a given lipid species detected in samples of TolC-mutant strain (n=6) by the average peak area of the same lipid species detected in samples of WT strain (n=6) (Mann-Whitney unpaired analysis, $p < 0.05$).

Compound	p TolC FGM vs WT FGM	p TolC SGM vs WT SGM	FC TolC vs WT FGM	FC TolC vs WT SGM
DGDG36:3	0.004	0.02	6.7	2.4
MGDG36:3	0.004	0.5	6.6	1.2
MGDG36:4	0.004	0.06	4.0	1.8
DGDG34:1	0.02	0.004	2.8	3.1
SQDG34:3	0.01	0.1	2.8	1.6
DGDG32:0	0.01	0.007	2.8	2.6
MGDG30:1	0.02	0.004	2.0	2.1
MGMG18:3	0.06	0.01	1.9	1.5
MGMG18:2	0.04	0.004	1.9	1.8
MGMG16:1	0.06	0.004	1.7	1.9

SQDG32:1	0.08	0.04	1.7	2.0
DGDG34:2	0.2	0.02	1.7	2.4
MGDG34:4	0.1	0.03	1.6	2.6
PG34:1	0.3	0.4	1.5	1.6
DGDG32:1	0.1	0.01	1.4	2.1
SQDG34:1	0.2	0.04	1.4	2.0
MGDG32:1	0.3	0.06	1.4	2.2
SQDG33:1	0.1	0.08	1.4	1.7
MGDG34:1	0.3	0.2	1.3	1.5
MGDG32:2	0.2	0.007	1.3	1.9
SQDG34:2	0.3	0.2	1.2	1.6
MGDG34:2	0.6	0.06	1.2	1.7
MGDG33:1	0.2	0.03	1.2	2.0
MGDG32:3	0.5	0.3	1.2	0.5
MGDG34:3	0.5	0.1	1.2	1.4
DGDG32:2	0.5	0.02	1.1	1.8
MGDG33:2	0.6	0.3	1.1	1.3
MGMG16:0	0.6	0.9	1.1	1.3
DGDG33:2	0.8	0.3	1.1	1.3
SQDG34:0	0.8	0.3	1.0	2.0
SQDG32:0	0.8	1.0	1.0	1.0
PG34:2	1.0	0.3	1.0	1.6
MGDG33:3	1.0	0.8	1.0	1.0
DGDG34:3	1.0	0.1	0.94	1.6
MGDG32:0	1.0	0.1	0.94	0.78
DGDG34:4	0.9	0.02	0.9	2.3
SQDG33:0	1.0	0.8	0.88	1.3
PG34:3	0.6	0.3	0.84	1.4
DGDG33:3	0.3	0.5	0.81	1.2

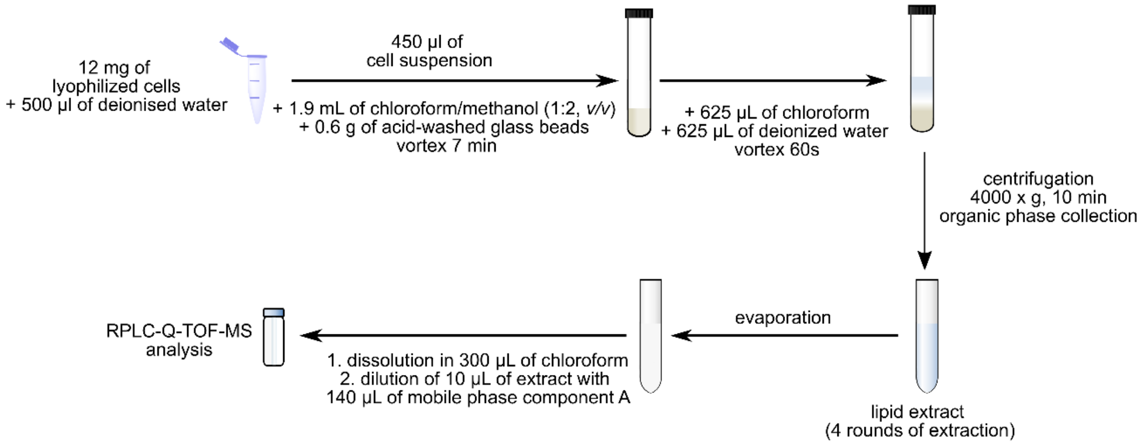

Figure S1. Analytical workflow used for the lipidomic analysis of *Synechocystis* sp. PCC 6803 cells.

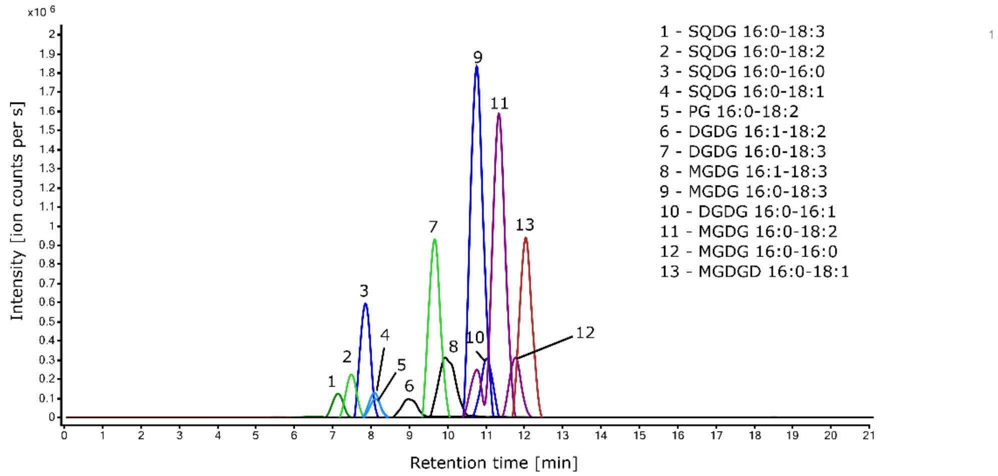

Figure S2. Representative ion chromatogram of the most abundant lipid species of *Synechocystis* sp. PCC 6803 wild-type.

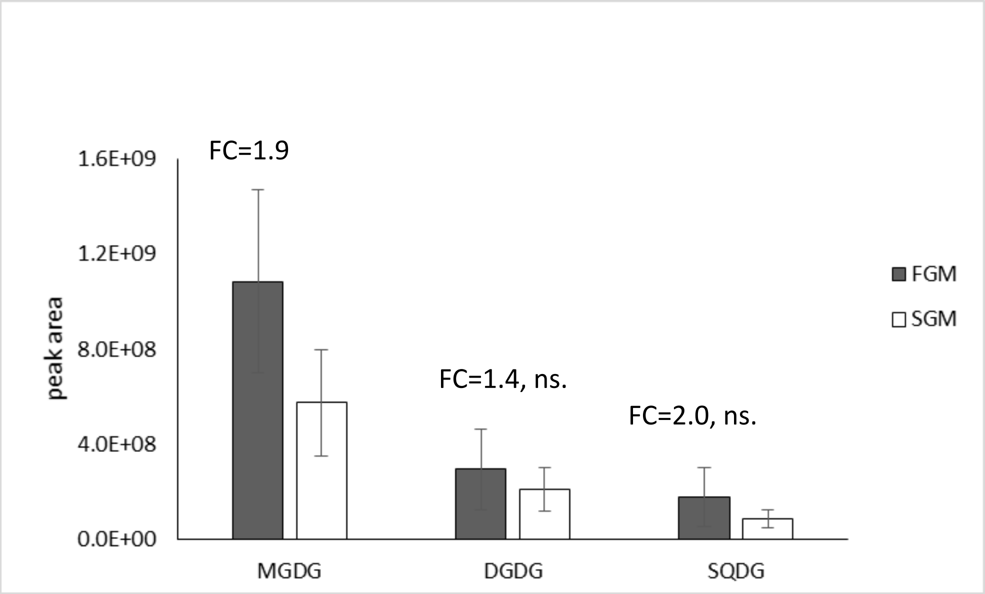

Figure S3. The total content of lipids among MGDG, DGDG and SQDG classes of the *Synechocystis* sp. PCC 6803 wild-type strain cultivated in slow- (SGM, white bars) or fast-growth mode (FGM, gray bars). Error bars indicate standard deviation corresponding to 6 independent biological replicates. FC – fold change. * $P < 0.05\%$; n.s.: not significant. (Mann-Whitney unpaired analysis, $p < 0.05$)

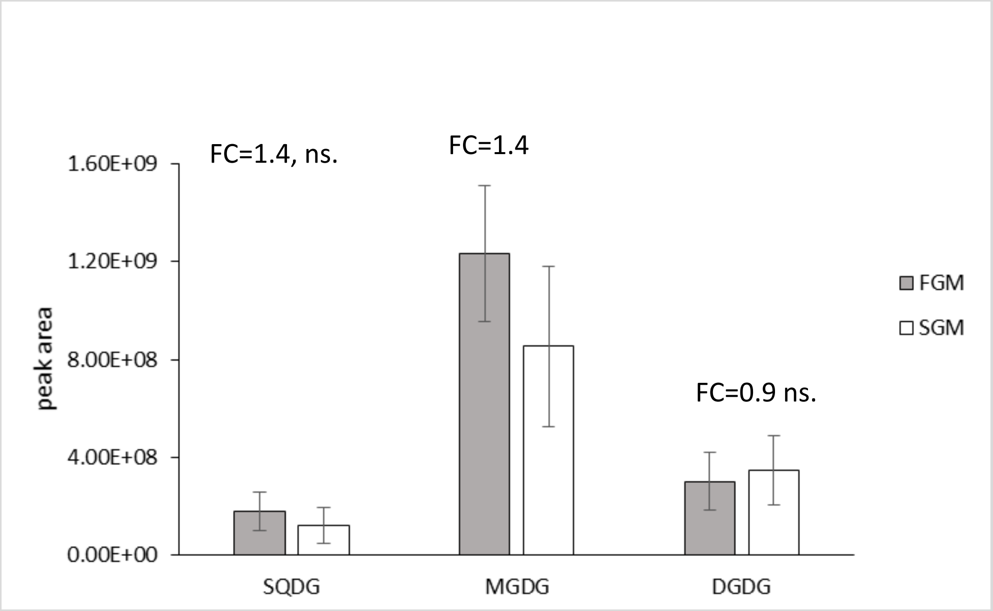

Figure S4. The total content of lipids among MGDG, DGDG and SQDG classes of the *Synechocystis* sp. PCC 6803 *tolC*-mutant strain cultivated in slow- (SGM, white bars) or fast-growth mode (FGM, gray bars). Error bars indicate standard deviation corresponding to 6 independent biological replicates. FC – fold change. ns.: not significant. (Mann-Whitney unpaired analysis, $p < 0.05$).
